# HspB1 phosphorylation regulates its intramolecular dynamics and mechanosensitive molecular chaperone interaction with filamin C

**DOI:** 10.1126/sciadv.aav8421

**Published:** 2019-05-22

**Authors:** Miranda P. Collier, T. Reid Alderson, Carin P. de Villiers, Daisy Nicholls, Heidi Y. Gastall, Timothy M. Allison, Matteo T. Degiacomi, He Jiang, Georg Mlynek, Dieter O. Fürst, Peter F. M. van der Ven, Kristina Djinovic-Carugo, Andrew J. Baldwin, Hugh Watkins, Katja Gehmlich, Justin L. P. Benesch

**Affiliations:** 1 Department of Chemistry, Physical and Theoretical Chemistry Laboratory, University of Oxford, South Parks Road, Oxford OX1 3QZ, UK.; 2 Division of Cardiovascular Medicine, Radcliffe Department of Medicine and British Heart Foundation Centre of Research Excellence Oxford, University of Oxford, Headington, Oxford OX3 9DU, UK.; 3 Biomolecular Interaction Centre and School of Physical and Chemical Sciences, University of Canterbury, Christchurch 8140, New Zealand.; 4 Department of Chemistry, Durham University, South Road, Durham DH1 3LE, UK.; 5 Department of Structural and Computational Biology, Max F. Perutz Laboratories, University of Vienna, Campus Vienna Biocenter 5, A-1030 Vienna, Austria.; 6 Department of Molecular Cell Biology, Institute for Cell Biology, University of Bonn, D53121 Bonn, Germany.; 7 Department of Biochemistry, Faculty of Chemistry and Chemical Technology, University of Ljubljana, Večna pot 113, 1000 Ljubljana, Slovenia.; 8 Institute of Cardiovascular Sciences, University of Birmingham, Birmingham B15 2TT, UK.

## Abstract

Mechanical force–induced conformational changes in proteins underpin a variety of physiological functions, typified in muscle contractile machinery. Mutations in the actin-binding protein filamin C (FLNC) are linked to musculoskeletal pathologies characterized by altered biomechanical properties and sometimes aggregates. HspB1, an abundant molecular chaperone, is prevalent in striated muscle where it is phosphorylated in response to cues including mechanical stress. We report the interaction and up-regulation of both proteins in three mouse models of biomechanical stress, with HspB1 being phosphorylated and FLNC being localized to load-bearing sites. We show how phosphorylation leads to increased exposure of the residues surrounding the HspB1 phosphosite, facilitating their binding to a compact multidomain region of FLNC proposed to have mechanosensing functions. Steered unfolding of FLNC reveals that its extension trajectory is modulated by the phosphorylated region of HspB1. This may represent a posttranslationally regulated chaperone-client protection mechanism targeting over-extension during mechanical stress.

## Teaser

The molecular chaperone HspB1 regulates the biomechanical extension of the heart muscle protein filamin C upon stress.

## INTRODUCTION

Mechanosensitive proteins withstand cycles of force-induced structural changes and hence have a pliable “native” state. This is critical for their function, whether to provide elasticity to cells and tissues, open and close a channel in the membrane, or reveal cryptic or force-enhanced binding sites in mechanotransductive signaling pathways (*1*). Excessive force, however, can lead to loss of function due to unfolding and subsequent formation of deleterious aggregates that overburden the capacity of the protein quality control network (*2*). These pathological deposits of intracellular proteins underlie a variety of muscle myopathies (*3*, *4*). To counter the effects of misfolding in general, cells invest in molecular chaperones. The frequent association of one class of chaperone in particular, the adenosine triphosphate (ATP)–independent small heat shock proteins (sHsps, HspBs) (*5*), with the cytoskeletal support network (*6*, *7*) and muscle contractile machinery (*8*) suggests a role for mechanosensitivity in sHsp-client binding.

**Fig. 1:**
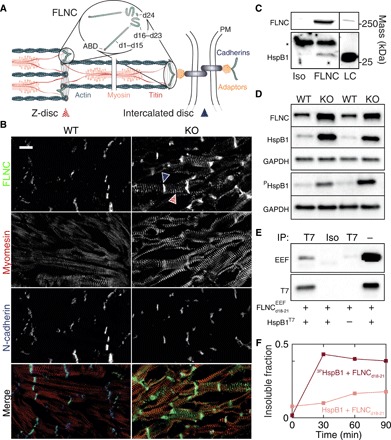
FLNC and HspB1 are up-regulated in MLP KO mouse heart and interact via domains 18 to 21 in a phosphorylation-dependent manner. (**A**) Schematic of FLNC composition and localization in striated muscle at the Z-disc and intercalated disc. Gray lines, multiple Z-disc proteins, including α-actinin, desmin, etc. Adaptors: talin, vinculin, catenins, etc. PM, plasma membrane. ABD, actin-binding domain of FLNC. Not pictured: possible localization at costameres. (**B**) Frozen sections of ventricular tissue from WT and MLP KO mice. Sections were stained for FLNC and counterstained to visualize sarcomeres (myomesin) and intercalated discs (N-cadherin). Scale bar, 10 μm. FLNC is primarily localized at intercalated discs and sarcomeric Z-discs (blue and red striped arrows, respectively) and is more abundant in the DCM tissue. (**C**) Immunoprecipitation (IP) from MLP KO ventricular tissue lysate using an FLNC-specific antibody or isotype control and lysate control. Molecular masses from positions of markers. Asterisk indicates signal from antibody chains. HspB1 coprecipitates with FLNC, evidencing an interaction between the endogenous proteins. (**D**) Western blots from WT and MLP KO mouse heart homogenates for FLNC, HspB1, ^P^HspB1, and GAPDH (glyceraldehyde phosphate dehydrogenase) as a loading control. Both FLNC and HspB1 were up-regulated in MLP KO hearts, and HspB1 was phosphorylated at Ser^86^ (equivalent to human Ser^82^). (**E**) Recombinant EEF-tagged FLNC_d18–21_ mixed with T7-tagged HspB1 and immunoprecipitated. T7 antibody (but not an isotype control) pulled down both HspB1 and FLNC_d18–21_, indicating binding between the recombinant proteins. Neither protein is pulled down when T7-HspB1 is omitted from the sample. (**F**) Pelleted fraction of mixtures of recombinant FLNC_d18–21_ and WT or ^3P^HspB1 over the course of 90-min incubation at 25°C. Measurements by densitometry of SDS–polyacrylamide gel electrophoresis (PAGE).

One potential interaction is between HspB1 (also known as Hsp27 in humans) and filamin C (FLNC; also known as γ-FLN; ABP-L; FLN2). Filamins, of which there are three paralogs in adult humans, are large modular proteins that play key roles in signaling pathways, cytoskeletal organization, cellular motility, and differentiation (*9*). They cross-link cytoskeletal actin into branched three-dimensional networks and are components of adhesion assemblies. FLNC, found almost exclusively in striated muscle, associates with thin filaments of sarcomeric actin at the myofibrillar Z-discs and intercalated discs (Fig. 1A) (*10*). Alterations in the human *FLNC* gene can lead to severely impaired myoarchitecture and deposits of large protein aggregates and were initially linked to skeletal myopathies (designated “filaminopathies”), with some carriers also displaying cardiac abnormalities (*11*). Recently, the range of known disease-linked *FLNC* alleles has expanded in association with various cardiac pathologies, often without skeletal manifestation (*12*, *13*). HspB1 is also prevalent in aggregates from patients with filaminopathy (*14*) and at sarcomeric lesions with FLNC (*15*), suggesting a possible association during stress.

Human filamins are ≈280-kDa homodimers, each monomer consisting of an N-terminal actin-binding domain followed by 24 immunoglobulin (Ig)–like domains (d1 to d24), the last of which mediates dimerization (Fig. 1A). The distal region (d16 to d24) encompasses the binding sites for most filamin partners (*9*), which appear to include HspB1, because this interaction was first detected in yeast two-hybrid assays using the segment d19–d21 of FLNC (*16*). The distal region of filamin A (FLNA) can sense mechanical cues by partially extending under force to reveal cryptic binding sites (*17*). FLNC contains a large insertion within d20, and less is known about its structure and the molecular basis of its roles. Evidence that FLNC can sense local force includes functionality that necessitates a degree of plasticity in response to tension (*18*), 73% sequence identity and overlapping roles and binding partners with FLNA (*19*), the ability to protect myofibrillar structural integrity (*20*), and differential in-cell labeling of shielded cysteines with applied force (*21*).

Each HspB1 monomer is composed of a conserved core α-crystallin domain (ACD) flanked by a short flexible C-terminal region, which makes intra-oligomer contacts, and a longer N-terminal domain (NTD) (*5*). The NTD also confers oligomeric stabilization, with phosphorylation at Ser^15^ and Ser^78^ or Ser^82^ causing a shift from a large polydisperse ensemble to smaller oligomeric species, including dimers (*22*, *23*). HspB1 is phosphorylated in cells and tissues in response to stretch, inducing translocation to the Z-discs (*24*, *25*) and to regions of increased traction force within the cytoskeleton (*26*), suggesting that phosphorylation may trigger or modulate its interactions with mechanosensitive clients such as FLNC at these sites.

To investigate the impact of phosphorylation on HspB1 intra-oligomer contacts and its interaction with FLNC, we used experimental approaches spanning the tissue to atomic levels. We validated the proposed interaction between FLNC and HspB1 and noted up-regulation and partial colocalization of both proteins, including the phosphorylated form of HspB1, in biomechanically challenged mouse hearts. We found that a segment of the NTD just downstream of the ACD in HspB1 adopts a range of conformations and determined how they are modulated by phosphorylation to trigger oligomer dissociation. A peptide segment from this flexible region binds multiple FLNC Ig domains. Performing coulombically steered unfolding in the gas phase as a proxy for the mechanical forces FLNC experiences in vivo allowed us to probe features of the interaction that are invisible at rest. This revealed that phosphorylated HspB1 inhibits a partially extended form of FLNC from further unfolding. Our results support a force-resistant binding mode between a molecular chaperone and a mechanosensitive target that is modulated by phosphorylation and may be important for cardiac function upon myoarchitectural perturbation.

## RESULTS

### FLNC and HspB1 are up-regulated and interact in biomechanically stressed mouse hearts

We sought to test the association between HspB1 and FLNC in cardiac tissue. To examine the effect of impaired cardiac biomechanics on FLNC expression and localization, we first used muscle LIM protein (MLP) knockout (KO) mice. MLP, like FLNC, is specific to striated muscle, and the phenotype of mice lacking MLP reproduces the morphology of dilated cardiomyopathy (DCM) and heart failure in humans (*27*). Frozen sections of ventricular tissue from wild-type (WT) and MLP KO mice were stained for FLNC and counterstained to visualize sarcomeric structure (myomesin) and intercalated discs (N-cadherin) (Fig. 1B). FLNC was located at intercalated discs and Z-discs at greater abundance in MLP KO hearts based on fluorescence intensity. This is consistent with previously reported FLNC localization (*10*) and with mechanosensitivity, given the role of intercalated discs and Z-discs as force-bearing sites. A proportion of HspB1 was coimmunoprecipitated together with FLNC from lysates of MLP KO ventricular tissue, indicating an endogenous interaction (Fig. 1C). Western blotting from WT and MLP KO mouse heart lysates confirmed that FLNC was up-regulated in the diseased tissue relative to WT (Fig. 1D). HspB1 was also up-regulated, including its phosphorylated form (at S^86^, analogous to human S^82^, the most prevalently modified site; mouse HspB1 does not contain a phosphosite equivalent to human S^78^; Fig. 1D).

We then asked whether FLNC and HspB1 are also up-regulated in WT hearts under biomechanical stress conditions. We subjected WT mice to transverse aortic constriction (TAC), an established procedure that results in pressure overload–induced cardiac hypertrophy and heart failure. TAC mouse hearts displayed higher levels of FLNC and HspB1 than sham WT hearts, as measured by Western blot (fig. S1A). Chronic treatment of WT mice with isoprenaline/epinephrine (IsoPE), increasing blood pressure and heart rate through adrenergic stimulation (*28*), also resulted in up-regulation of both proteins (fig. S1B). Immunofluorescence of ventricular tissue from TAC- and IsoPE-treated mouse hearts showed FLNC at intercalated discs and Z-discs as in the MLP KO model (fig. S1C). We also observed varying degrees of colocalization of HspB1 with FLNC in all three groups (MLP KO, TAC, and IsoPE), with comparatively weak HspB1 signal in controls (fig. S1D).

Next, to validate the interaction in vitro, we expressed EEF-tagged human FLNC domains 18 to 21, encompassing the apparent HspB1 binding site (*16*), and full-length human HspB1 attached to a T7 tag. Pulling down the HspB1 protein with the T7 antibody coimmunoprecipitated FLNC_d18–21_ (Fig. 1E), confirming a direct interaction between these proteins. To investigate the effect of HspB1 phosphorylation on the interaction, we expressed WT human HspB1 and ^3P^HspB1, a triple mutant containing phosphomimetic Ser to Asp substitutions at residues 15, 78, and 82. Upon incubation with FLNC_d18–21_ in vitro, we noticed that mixtures accumulated insoluble protein aggregates, while isolated proteins remained soluble. In a 2:1 mixture of the chaperone to the FLNC fragment, both proteins migrated to the pelleted fraction over time, an effect that was substantially increased by phosphomimicry (Fig. 1F and fig. S2). The results support an interaction between HspB1 and FLNC_d18–21_ to form large complexes that can be modulated by phosphorylation of HspB1.

### Phosphomimicry reduces oligomer size and coincides with disorder in the N-terminal region of HspB1

**Fig. 2:**
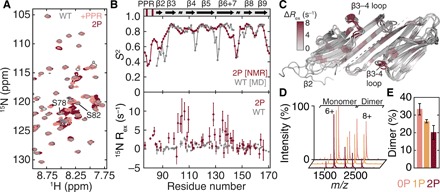
HspB1_77–171_undergoes dynamical changes leading to partial dissociation upon phosphomimicry. (**A**) Overlaid ^1^H-^15^N heteronuclear single-quantum coherence (HSQC) spectra of HspB1_84–171_ (gray), HspB1_77–171_ (pink), and ^2P^HspB1_77–171_ (maroon). More signals are present in the region 7.8 to 8.6 ppm upon phosphomimicry. (**B**) N-H order parameters (*S*^2^) derived using assigned backbone chemical shifts from ^2P^HspB1_77–171_ (maroon) and from MD simulations of HspB1_84–171_ (gray) confirm that the PPR and β2 strand are highly dynamic (top). A plot of *R*_ex_, a qualitative metric of microsecond-millisecond motions probed by ^15^N CPMG relaxation dispersion, for HspB1_84–171_ (gray) and ^2P^HspB1_77–171_ (maroon) reveals a significant increase in dynamics in residues 95 to 110 (bottom). Secondary structure elements from PDB 4MJH are indicated. *R*_ex_ data are means ± SD. (**C**) Snapshots from a 1-μs MD simulation of HspB1_84–171_ showing the complete dissociation of a β2 strand. Residues are colored according to *R*_ex_ [from (B)], showing the region of increased dynamics to be in the β3-β4 loop on the side of the ACD. (**D** and **E**) Native mass spectra and stoichiometric quantification of HspB1_77–171_ WT (pink), 1P (^P^Ser^82^, yellow), and 2P (^P^Ser^78^-^P^Ser^82^, maroon). The construct populates a monomer-dimer equilibrium and phosphomimicry shifts the oligomeric state toward the monomer. Data are means ± SD.

Full-length HspB1 assembles into large, dynamic, and polydisperse oligomers that compromise its investigation at high resolution (*5*). To interrogate the regulatory role of phosphorylation, we therefore built on our previous construct of the structured ACD (HspB1_84–171_), which does not assemble beyond a dimer but retains chaperone activity (*29*). We incorporated a small part of the NTD just upstream of the β2 strand, which we term the “principal phosphorylation region” (PPR), as it includes phosphosites Ser^78^ and Ser^82^ (HspB1_77–171_). We also mutated these sites to mimic phosphorylation events (^2P^HspB1_77–171_). Two-dimensional nuclear magnetic resonance (NMR) spectra of each of the three constructs featured well-dispersed resonances (Fig. 2A and fig. S3A), evidencing folded dimer structures. The HspB1_84–171_ and HspB1_77–171_ spectra overlay closely, with additional peaks in the region diagnostic of disorder (7.8 to 8.6 ppm in the ^1^H dimension) arising in the longer construct from the additional N-terminal residues (Fig. 2A). To examine the impact of phosphorylation on the PPR, we compared the NMR spectra of HspB1_77–171_ and ^2P^HspB1_77–171_. We noted clear differences, particularly intensified signal in the disordered region for the phosphomimic, consistent with an increase in flexibility (Fig. 2A and fig. S3A). Chemical shift–based order parameters and secondary structure propensities indicated a high degree of backbone flexibility and a low amount of secondary structure for the β2 strand and the PPR (Fig. 2B).

To visualize the structural fluctuations of β2, we performed a 1-μs molecular dynamics (MD) simulation of the HspB1 ACD starting from a structure [Protein Data Bank (PDB): 4MJH] in which the β2-strand is intramolecularly hydrogen-bonded to β3. We found that over the course of the simulation, β2 fluctuated considerably, readily losing its secondary structure and detaching from the rest of the ACD (Fig. 2C). Calculation of the order parameters from the simulation reveals good correspondence with that from our NMR data (Fig. 2B) and is consistent with the disorder observed by others (*30*).

We next looked at chemical shift perturbations (CSPs) in the HspB1 ACD upon phosphomimicry. These were widely dispersed but small in magnitude (fig. S3B), supporting similarity of the ACD structures. To probe for effects on internal dynamics, we recorded ^15^N spin relaxation measurements that monitor both picosecond-nanosecond and microsecond-millisecond motions. Whereas the fast dynamics were unaffected (fig. S3C), residues 95 to 110 (including the loop between β3 and β4) in the phosphomimic displayed evidence of conformational exchange on the slower timescale (Fig. 2B and fig. S3, D and E). We reasoned that this could indicate transient sampling of a lowly populated state involving contact between the PPR and the ACD. The major state would then contain an unbound PPR, consistent with the small CSPs between HspB1_84–171_ and HspB1_77–171_ for residues 102 to 110, lack of any conformational exchange slow enough to give rise to multiple peaks, and flexibility of the N-terminal residues in solution (Fig. 2B).

The absence of notable broadening in the NMR spectra as a function of increasing HspB1_77–171_ concentration suggested that the contacts made by the PPR regulate intradimer rather than interdimer binding. To test this hypothesis, we recorded mass spectra under conditions preserving noncovalent interactions. Applied to full-length HspB1, this method has revealed a shift toward small species upon phosphomimicry, with predominant monomer and accompanying dimer at low micromolar concentration (*23*). The new data showed that in the same concentration range, HspB1_77–171_ populates a monomer-dimer equilibrium that is similarly shifted toward dissociation upon incorporation of one and two phosphomimics (Fig. 2, D and E). The absence of higher-order oligomers indicates that appreciable intermolecular binding of the PPR is unlikely in the context of the truncated construct, and the agreement between the full-length and truncation results supports the use of our construct in probing the link between phosphorylation-induced dynamics and quaternary organization. The combined NMR and mass spectrometry (MS) data are therefore consistent with phosphorylation modulating an order-to-disorder transition by β2 and the PPR that leads to oligomer dissociation via rearrangement of intradimer contacts.

### The PPR of HspB1 interacts with a pocket in the ACD

**Fig. 3:**
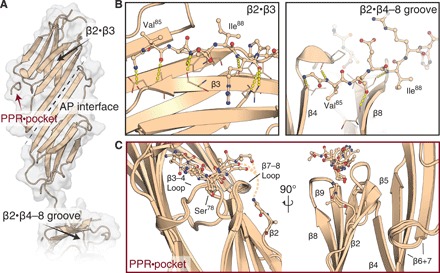
Crystal structure of the HspB1 ACD in complex with a partial peptide mimic of the PPR. (**A**) HspB1 ACD dimer with peptide PPR mimic bound. Dotted line indicates the antiparallel interdimer interface. (**B**) Areas indicated by black arrows in (A) showing two locations of the β2 strand: bound to β3 (left) or dissociated from the ACD and reaching across the β4-β8 groove of a neighboring monomer (right). (**C**) PPR binding [red arrow in (A)], shown as an overlay of the four peptide-bound monomers in the asymmetric unit. Whereas the ACDs overlay closely, the peptides appear heterogeneous and flexible. Rotated view shows β2 strand divergences, sometimes arcing toward the peptide binding site and in one instance partially modeled as continuation of the peptide.

We next carried out crystallographic trials, using a strategy of mixing peptide mimics of the PPR, varying in length and degree of phosphorylation, with a well-ordered ACD construct (HspB1_84–170_). This led to a structure of HspB1_84–170_ bound to ALSRQL^P^SSGVSEI (residues 76 to 88 phosphorylated at Ser^82^), solved by molecular replacement in space group *P*12_1_1, that contains four dimers in the asymmetric unit (table S1 and fig. S4A). The monomers within each dimer differ at their N-terminal ends: The β2 strand of one is intramolecularly hydrogen-bonded to its own β3 strand, whereas in the other these residues are extended and stabilized by contacts with a β4-β8 groove of a neighboring dimer (Fig. 3, A and B). This groove also acts as a binding pocket for an intrasubunit interface within the C-terminal region (fig. S4B) (*29*).

Upon refinement of the ACD, the 2*F*_o_-*F*_c_ and difference density maps displayed a clear unmodeled feature occupying a pocket between the β3-β4 and β8-β9 loops, which lies on the side of the subunit close to the dimer interface. This feature was only present adjacent to subunits with intramolecularly bound β2 strands (fig. S4C). We were able to build peptides into this density but only accommodating subsets of the 11 possible residues, with their side-chain densities remaining poor in contrast to the ACD (see Materials and Methods). An ensemble of the four peptide-bound ACDs provides the best overview of the interaction available from these data (Fig. 3C). The binding site aligns with the dynamical changes we observed by NMR (Fig. 2, B and C). Our results reveal how the β2 strand and PPR assume heterogeneous conformations. Although predominately disordered in solution (Fig. 2, B and C), this region is also capable of making intramonomer associations with the β3 strand and stretching to the pocket between β3-β4 and β8-β9 loops (Fig. 3, B and C), as well as intersubunit interactions by occupying a β4-β8 groove in a neighboring dimer (Fig. 3B). While it is not clear what the relative populations of these states are in the context of the full-length HspB1, they are consistent with multiple NTD conformations contributing to maintaining sHsp assembly (*5*). Our combined data reveal that this heterogeneity is modulated by phosphorylation, resulting in changes in the stabilities of the interfaces that are likely responsible for shifting the equilibrium away from large oligomers toward smaller species.

### HspB1 residues 80 to 88 bind multiple domains within FLNC

**Fig. 4:**
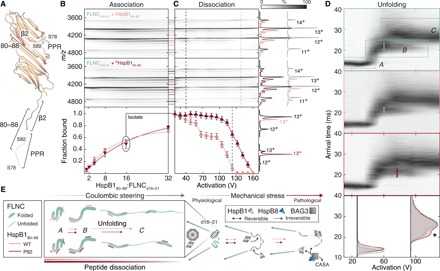
^(P)^HspB1_80–88_ and FLNC_d18–21_ form a complex that extends in a phosphorylation-dependent manner. (**A**) The location of HspB1 region 80 to 88, the FLNC_d18–21_ binding epitope used as a peptide in subsequent IM-MS experiments, is indicated on each subunit of the HspB1_84–170_ dimer structure. (**B**) Contour plots of native mass spectra of FLNC_d18–21_ obtained upon titrating the unphosphorylated (top) and phosphorylated (middle) peptides. Association of multiple peptides is observed, based on the emergence of new peaks in the mass spectra, appearing as additional horizontal streaks in the plot. Pink lines denote peptide-bound FLNC; line thickness designates charge series and hence the same stoichiometry. Binding curves, fit using standard methods and assuming up to three equivalent binding sites, reveal similar affinities of each peptide to FLNC_d18–21_ (bottom). Data are means ± SEM. (**C**) Dissociation of the peptide was affected by activation in the gas phase, with the phosphorylated peptide remaining bound to higher potentials than the unphosphorylated equivalent. Breakdown curves (derived from tandem MS data, see fig. S5) reveal clear differences between the peptides—shown are continuous single experiments, with error bars representing one SD as calculated from repeats. Mass spectra underlying dashed lines in (B) are projected to the right. At 40 V, binding differences are negligible, but at 140 V, all of the unphosphorylated peptide has been ejected but the phosphopeptide has not. (**D**) Coulombically steered unfolding of the 13+ charge stage of FLNC_d18–21_ unbound, bound to one HspB1_80–88_, and bound to one ^P^HspB1_80–88_. Bottom, overlaid arrival time distributions at 30 and 90 V, highlighting the persistence of the intermediate state with bound phosphopeptide (*). Lines on unfolding plots designate the activation required to transition half of the intermediate to the final state. (**E**) Schematic of force-induced changes to FLNC underlying the unfolding plots and relation to HspB1 peptide binding. The right panel contextualizes mechanical stress and the full-length proteins.

To isolate a stable HspB1-FLNC_d18–21_ complex and localize the binding within HspB1, we performed native ion mobility MS (IM-MS) experiments. FLNC_d18–21_ displayed an envelope of peaks centered on low charge states, and a collision cross-section (CCS) of 37.2 ± 0.8 nm^2^ (fig. S5A). This is consistent with a compact rather than linear architecture (fig. S5B), suggesting an inherently extensible conformation for this FLNC segment, wherein noncovalent interdomain interactions would be susceptible to rupture under force. Next, we mixed FLNC_d18–21_ with either HspB1_84–171_ or peptides mimicking the PPR, because this region is phosphorylated in the MLP KO mice (Fig. 1C) and generally upon biomechanical stress (*26*) and becomes exposed thereafter. This strategy of using segments of HspB1 meant that we could avoid the problems of insolubilization of the full-length protein upon interaction with FLNC _d18–21_, as well as generally mitigate the confounding effects associated with the chaperone’s intrinsic polydispersity. We were able to detect complexes between FLNC_d18–21_ and up to two copies of a peptide-containing part of the PPR, HspB1_80–88_ (or its phosphorylated counterpart ^P^HspB1_80–88_) (Fig. 4A and fig. S5D). Complexes were not observed in mixtures of FLNC_d18–21_ with HspB1_84–171_ (fig. S5C), suggesting that HspB1 residues 80 to 83 are critical, or with negative control peptides (fig. S5D), revealing a specific interaction with this segment of HspB1.

To measure the association of ^P^HspB1_80–88_ and HspB1_80–88_ to FLNC_d18–21_, we recorded mass spectra at increasing peptide concentrations, now observing up to three copies bound (Fig. 4B and fig. S5F). By quantifying the relative abundances of FLNC_d18–21_ and the peptide-bound species, we could determine binding affinities (Fig. 4B, lower panel, and fig. S4G). We obtained similar dissociation constants (*K*_D_s) of 130 ± 17 μM (HspB1_80–88_) and 110 ± 10 μM (^P^HspB1_80–88_), indicating weak binding that is not affected substantially by phosphorylation. These findings contrast with the notable effect of HspB1 phosphomimicry on the interaction of full-length chaperone with the FLNC fragment (Fig. 1E). However, they can be rationalized by phosphorylation increasing the effective concentration of the binding region by modulating its conformational state (Fig. 2B and fig. S3) and leading to dimer (Fig. 2D) and oligomer disassembly (*22*, *23*). To localize binding of HspB1_80–88_ to domains with Ig folds or to the d20 insertion (which is possibly disordered), we prepared a modified FLNC construct lacking the 82 insertion residues (FLNC_d18–21,ΔI_). This construct also binds multiple HspB1 peptides (fig. S5E), indicating that HspB1 recognizes folded Ig domains of FLNC.

### Phosphorylation modifies the extension of the FLNC-HspB1 complex

Having found marginal difference in binding affinities between the phosphorylated and unphosphorylated HspB1 peptides with FLNC_d18–21_ at equilibrium, we next sought to probe the strength of this interaction under the application of force. The motivation for this stemmed from the observation that the filamin distal region (Fig. 1A) undergoes extension in vivo (*21*), with FLNA domains even predicted to be able to unfold fully before breakage of the dimer interface, at physiologically relevant forces and loading rates (*18*, *31*). Given the weakness of the FLNC-HspB1_80–88_ complexes (>100 μM, see above), and consequently their predicted low abundance at the concentrations compatible with force spectroscopy, we turned instead to an IM-MS alternative as a proxy for mechanical stress. Specifically, we performed experiments in which protein ions at a designated mass/charge (*m*/*z*) ratio are isolated in the mass spectrometer and activated selectively by collisions with inert gas, leading to conformational perturbations and chain extension steered by the coulombic repulsion between like charges (*32*).

Upon extending the HspB1_80–88_:FLNC_d18–21_ complex by ramping the activation potential, we observed a gradual reduction in the abundance of peptide-bound FLNC_d18–21_, indicating dissociation of the peptide (Fig. 4C). Notably, phosphorylation of HspB1_80–88_ greatly increased the energy required to remove the peptide from FLNC: At 140 V, all unphosphorylated HspB1_80–88_ had dissociated, while ^P^HspB1_80–88_-bound FLNC persisted until 180 V (Fig. 4C, right). To quantify this differential dissociation behavior while avoiding potential cross-talk between charge states, we selected the 13+ ion of FLNC_d18–21_ bound to a single peptide in the mass spectrometer and performed analogous activation ramps (fig. S4H). This allowed us to extract the fraction of FLNC_d18–21_ associated with each peptide as a function of activation (Fig. 4C, lower). The breakdown curve for the FLNC_d18–21_:HspB1_80–88_ complex revealed two inflection points, at ≈55 and ≈110 V. This suggests that HspB1_80–88_ associates with FLNC_d18–21_ in (at least) two locations, consistent with the observation of multiple peptides binding in our titration experiments (Fig. 4B). ^P^HspB1_80–88_ dissociated less readily from FLNC_d18–21_, and the breakdown curve features only one inflection point at ≈130 V.This suggests that phosphorylation increases both the selectivity and strength of interaction of HspB1 with FLNC during coulombically steered extension.

To explore the phosphorylation-dependent differences between the FLNC-HspB1 peptide complex observed during extension (Fig. 4C) but not at rest (Fig. 4B), we used IM to examine the unfolding trajectory of FLNC_d18–21_. Over the range of activation potentials that dissociate HspB1 peptides from FLNC_d18–21_ (Fig. 4C), FLNC_d18–21_ extends from the initial folded state (*A*) through an intermediate (*B*) to reach a final state (*C*) (Fig. 4D, upper). To validate this pathway in the context of the biomechanics of FLNC, we asked whether we could correlate the transitions measured by IM to biologically functional events. Using shorter, two-domain FLNC constructs to increase the resolution of discrete unfolding transitions, we removed the first β-strand from domain 20, which shields a binding site for signaling partners at cell adhesions that becomes exposed upon mechanical force (fig. S6A) (*19*, *33*). This deletion resulted in abolishment of the intermediate, meaning that a key step in the biological unfolding pathway is clearly reported by our gas-phase approach (fig. S6B). For further validation, in the longer construct we used to assess binding to the HspB1 peptide (FLNC_d18–21_), we introduced a more conservative perturbation. Rather than delete the entire β-strand, we engineered into it two substitutions (E2136R and I2138E). Again, the coulombically steered unfolding pathway was significantly affected: while the stability of *A*, based on the potential required to transition to *B*, was unaffected, *B* was destabilized with respect to unfolding further to *C* (fig. S6C). These data therefore lead us to conclude not only that the gas-phase unfolding approach is biomechanically relevant but also that the FLNC_d18–21_ transition *B-C* involves disruption of contacts made by the mechanically functional d20 β-strand.

The same three states were seen for FLNC_d18–21_ with either peptide bound (Fig. 4D). In all cases, unfolding commenced at a lower potential than necessary for peptide dissociation. Therefore, HspB1 does not exclusively bind the regions within FLNC_d18–21_ that extend first, because unfolding of a ligand-bound domain will coincide with ejection of the ligand (*34*). The stability of *A* was not altered by either peptide. State *B*, however, required more activation to transition to *C* when bound to ^P^HspB1_80–88_ (≈75 V) versus HspB1_80–88_ (≈60 V) (Fig. 4D). This difference was consistent across charge states and instrument conditions (fig. S7). The data point to the formation of interactions when the phosphopeptide-bound FLNC structure is activated, leading to its increased resistance to extension, and dissociation at higher potentials than the unmodified peptide (Fig. 4C). Given that the transition from *B* to *C* includes rupture of the β-strand swap, we hypothesize that a binding site of HspB1 is in the vicinity of this association. In sum, our data suggest a chaperone-target mechanism reliant on the recognition of native-like states but stabilization of unfolding intermediates, in a manner that is regulated by posttranslational modification.

## DISCUSSION

We have investigated how phosphorylation of HspB1 modulates its interaction with FLNC. Biophysical and structural studies reveal conformational heterogeneity in the β2 strand and the immediately upstream PPR, which encompasses the primary HspB1 phosphosite. These residues are largely disordered in our construct in solution, but are able to make transient intramolecular contacts with β3 and a pocket on the edge of the ACD in a manner that is regulated by phosphorylation. We observed an interdimer interaction between the β2 strand and the β4-β8 groove, also the location of a C-terminal interdimer interaction (*29*), in our crystal structure. This is consistent with alternative modes of occupancy for the β4-β8 groove observed in other vertebrate sHsps (*5*, *35*) and hints at intramolecular competition for the binding site in the context of the HspB1 oligomers, a mechanism recently proposed for another client interaction (*36*). The conformational plasticity may be the primary source of the modulation in chaperone activity associated with HspB1 phosphorylation, exposing binding sites both directly through intramolecular rearrangements and indirectly by causing oligomeric dissociation (*22*, *23*).

Similar heterogeneity of N-terminal residues has been observed in other sHsps and shown to regulate assembly, including in HspB5 and HspB6, both of which can co-assemble with HspB1 in vivo (*37*). The HspB5 β2 strand and residues immediately preceding it exist in multiple conformations, including bound to β3 both intra- (*38*) and intermonomer (*39*). Recent crystal structures of HspB6 show no resolved density for β2 and support disorder-to-order transitions upon interactions made by the N terminus (*40*). In both HspB5 and HspB6, these contacts are controlled by phosphorylation (*5*, *40*). Together, these observations indicate a mechanism of tunable function via phosphorylation-based modulation of local flexibility that alters structure at various levels of organization both within and between sHsp family assemblies.

In MLP KO mice, which display heart failure, we detected an interaction between HspB1 and FLNC and prevalent phosphorylation of HspB1 at Ser^82^. In the MLP KO model, as well as TAC- and IsoPE-treated WT mouse hearts, both FLNC and HspB1 were up-regulated and partially colocalized. These data evidence an interaction enhanced by biomechanical stress. They are consistent with previous reports of HspB1 up-regulation in DCM hearts (*41*, *42*), its phosphorylation in response to cell or tissue stretch (*24*, *26*), and its translocation to sarcomeric Z-discs following muscle exercise or mechanical loading (*8*, *15*, *25*). Within the proteins, we localized the binding in vitro to HspB1_80–88_ and FLNC_d18–21_. Residues 80 to 88 encompass parts of the HspB1 PPR and β2 strand, and we see binding of this region as a peptide to FLNC_d18–21_ in a multivalent fashion. While phosphorylation of HspB1 increased the association by promoting unbinding and flexibility of the PPR, it did not require the target to be unfolded to be recognized. This behavior may be facilitated by HspB1 and FLNC_d18–21_ both containing Ig folds such that chaperone-target interfaces mimic intramolecular interactions in HspB1.

Such interaction between an sHsp and a target in its native (rather than a misfolded) state is one of only a few observed directly (*36*, *40*, *43*). These tend to present low-affinity binding modes, in keeping with our *K*_D_ measurements of 130 and 110 μM for HspB1_80–88_ and ^P^HspB1_80–88_, respectively, to FLNC_d18–21_. Our NMR data support that, within HspB1, this segment has a high propensity for disorder. Weak interactions involving intrinsically disordered regions are overrepresented among temporally fluctuating processes (*44*), and within cardiomyocyte adhesion complexes, functional associations have been measured with *K*_D_s as weak as 290 μM and even 3 mM (*45*, *46*). A transient interaction could minimize the obstruction of folded FLNC domains from binding partners for normal function, e.g., signaling. Phosphorylation of HspB1 would then play a dual role, increasing the interaction rate by modulating availability of the HspB1 binding site through its release from oligomers while also enabling rapid sensing of unfolding FLNC during biomechanical stress.

Because a static affinity measurement will lack this mechanical unfolding component, and to be able to selectively target the low-affinity complex, we tested this hypothesis by mimicking strain on FLNC via extension in the gas phase (*32*). This approach steers using the coulombic repulsion between like charges, and electric field–induced protein motions have been shown to reflect functional trajectories (*47*). Although the free energies of unfolding transitions can be influenced by the lack of solvent in these experiments (*48*), the method provides a means of probing differences along the pathway. Our experiments revealed that FLNC_d18–21_ adopts a compact state, meaning that multiple interdomain interactions are likely. This coheres with predictions of its similarity to FLNA_d18–21_, whose semicompact L shape forms part of a propeller-like FLNA_d16–21_ region with β strand swapping between domain pairs (*17*, *49*). Upon extending FLNC domains, we consistently observed conformational change connected to a biologically relevant transition. Notably, when the HspB1 peptide was bound, initial FLNC_d18–21_ unfolding proceeded without its ejection, and an intermediate was stabilized by ^P^HspB1_80–88_ relative to HspB1_80–88_. The results are consistent with a phosphorylation-triggered function of HspB1 in regulating the extension of FLNC.

The degree of FLNC extension in vivo depends on various factors, including its dimer and actin interface strengths, because breakage of these would alter the strain borne by the protein along its length (*31*). While these interface strengths have not been reported for FLNC, reversible FLNA domain unfolding and actin unbinding are energetically similar, and each is more common than dimer rupture at physiological forces and low loading rates (*50*). It will be interesting to investigate whether the ^P^HspB1-FLNC interaction is energetically favored at a given loading rate, as documented for other mechanosensitive processes (*51*). This could concur with the increase in expression of both proteins that we observe in hearts of MLP KO mice, the most pronounced up-regulation in our study. Functional importance could take the form of myoarchitectural integrity maintenance, because these animals display severe defects in myofibrillar organization (*27*). FLNC may require chaperoning to function properly under stress and avoid adverse structural disruption. In this role, HspB1 binding appears to be preventative rather than a response to damage, lowering the risk of FLNC hyperextension and aggregation.

Nevertheless, critical force overburdens this system, triggering chaperone-assisted selective autophagy (CASA) to clear and degrade filamin (FLNA and FLNC reported) in muscle tissue. CASA involves the recognition of compromised filamin by the BAG3-HspB8 chaperone complex, releasing it from the Z-disc for autophagic degradation and subsequent triggering of a signaling cascade to stimulate filamin transcription (*52*). While HspB8 is the preferred binding partner of BAG3, additional sHsps may interact with this co-chaperone too (*53*). Future work will examine whether ^(P)^HspB1 attenuates mechanical unfolding of FLNC in vivo, and whether lowering levels of HspB1or inhibiting its phosphorylation affect FLNC aggregation and turnover. Colocalization of HspB1 with FLNC and BAG3 at skeletal muscle sarcomeric lesions (*15*) and in our mouse disease models, its ability shown here to bind both compact and extending filamin, and its known co-assembly with several sHsp paralogs support HspB1 as a potential key hub in muscle proteostasis (Fig. 4E).

Our results, in the context of these previous reports, point to a two-tier sHsp-mediated proteostasis mechanism for maintaining FLNC and the myoarchitecture, active at varying tensions. This is modulated by the cellular response to mechanical stress, via HspB1 phosphorylation, exposure of its FLNC binding site, and stabilization of the reputed mechanosensitive region of FLNC. Recent reports have detailed binding of chaperones to model proteins destabilized by the application of force (*54*, *55*). These findings suggest a physiological context for such a mechanism and may provide a new avenue for understanding filaminopathies and FLNC-linked cardiac-specific diseases.

## MATERIALS AND METHODS

### Animal experiments

Experimental procedures were performed in accordance with the UK Home Office guidelines (project licenses 30/2444 and 30/2977) and approved by respective institutional review board. Animals were housed in specific pathogen–free conditions, with the only reported positives on health screening over the entire time course of these studies being for *Tritrichomonas* sp. and *Entamoeba* spp. All animals were housed in social groups, provided with food and water ad libitum, and maintained on a 12-hour light/12-hour dark cycle (150 to 200 lux cool white light-emitting diode light, measured at the cage floor). MLP KO mice (*27*) were backcrossed with C57BL/6J for more than six generations before generating homozygous MLP KO mice and used at 3 months of age. Age-matched WT C57BL/6J were obtained from Harlan.

TAC surgery was performed as described (*56*) on male C57BL/6J mice of 2 to 3 months of age. Sham-operated animals (in which the aortic arch was dissected, but no suture was tied) served as controls. Adrenergic challenge treatment was done as described (*28*). Briefly, male C57BL/6J mice of 2 to 3 months of age were treated with isoprenaline hydrochloride and phenylephrine hydrochloride (IsoPE; both from Sigma-Aldrich, UK) at 15 mg kg^−1^ body weight per day each for 14 days in 0.9% saline via subcutaneously implanted micro-osmotic pumps (model 1002, ALZET). Pumps were implanted during a short surgical procedure carried out under general anesthesia. Control mice received pumps delivering 0.9% saline vehicle solution only and were monitored in the same way as adrenergic-challenged mice. Hearts were harvested 2 weeks after the interventions.

### Coimmunoprecipitation

T7-tagged purified recombinant HspB1 protein (1 μg) was added to 10 μg of EEF-tagged recombinant FLNC_d18–21_ in immunoprecipitation (IP) buffer [0.05% Triton X-100, 1% bovine serum albumin, protease inhibitors (Roche mini complete EDTA-free tablets) in phosphate-buffered saline (PBS)] and incubated for 1 hour, shaking at room temperature. This was followed by another 30-min incubation, shaking at room temperature after the addition of 0.5 μg of anti-T7 (69522, Novagen) or mouse IgG2b isotype (ab18457, Abcam) antibody. Next, 25 μl of protein G Sepharose resin (PC-G5, Generon) was added to each tube and incubated for 1 hour, shaking at room temperature. The beads were then washed four times with IP buffer, resuspended in 20 μl of 5× SDS sample buffer [312.5 mM tris-HCl (pH 6.8), 500 mM dithiothreitol (DTT), 10% SDS, 30% glycerol, 0.05% bromophenol blue], and boiled at 95°C for 5 min. Immunodetection was performed using the anti-T7 (69522, Novagen) or anti-EEF/anti-tubulin (YL1/2) (ab6160, Abcam) antibodies.

### Analysis of mouse hearts

Animals were sacrificed by cervical dislocation, and hearts were flushed with ice-cold PBS and snap-frozen in liquid nitrogen. Immunofluorescence from frozen tissue was performed as described (*45*) using FLNC antibody RR90 (*10*), pan-cadherin antibody (Sigma), and myomesin antibody B4 (Developmental Studies Hybridoma Bank). Immunoblotting from total protein extracts was performed as described (*45*) using anti-HspB1 (2442, Cell Signaling Technology), anti-^P^HspB1 (Ser^82^) (2401, Cell Signaling Technology), anti-GAPDH (glyceraldehyde phosphate dehydrogenase) (ABS16, Merck), and FLNC [R1899; MRC PPU (Medical Research Council Protein Phosphorylation and Ubiquitinylation Unit) Reagents and Services, University of Dundee] antibodies. IP was performed as described (*45*) using 5 μg of FLNC (R1899) antibody. Blotting for HspB1 (2442, Cell Signaling Technology) was performed using “VeriBlot” (ab131366, Abcam) as secondary horseradish peroxidase conjugate.

### Expression and purification of HspB1 constructs

A pET28d(+) vector encoding human HspB1 ACD (residues 84 to 171) was transformed into *Escherichia coli* BL21(DE3) cells (Agilent) and expressed and purified as described previously (*29*). For crystallography, residue K171 was deleted using a site-directed mutagenesis kit (Agilent) and HspB1_84–170_ was expressed and purified as described. HspB1_77–171_ and variants thereof were cloned into the same vector and expressed in the same manner, with both proteins containing a residual Gly-Ser overhang between the tobacco etch virus (TEV) protease recognition site and the beginning of the HspB1 sequence. Phosphomimic mutations S78E and S82D were introduced using site-directed mutagenesis, expressed according to the same protocol, and purified as follows: Cells were lysed using a cell press with added protease inhibitor cocktail (Roche). Lysate was centrifuged at 20,000*g* for 20 min, and the supernatant was filtered and loaded onto a HisTrap column in buffer [20 mM tris (pH 8), 150 mm NaCl, 20 mM imidazole, 5 mM β-mercaptoethanol (BME)]. Protein was eluted with a gradient to 500 mM imidazole, pooled, and dialyzed overnight with TEV protease into the loading buffer at room temperature. The sample was then exchanged into the same buffer with addition of 8 M urea by cycles of centrifugation and dilution using an Amicon concentrator with 3.5-kDa cutoff. The cleaved and unfolded protein was passed over a HisTrap column, and flow-through was collected and refolded by overnight dialysis at room temperature into 100 mM NaCl, 20 mM tris (pH 7.5). Protein was then concentrated, and DTT was added to a final concentration of 1 mM for storage at −20°C until use. For NMR experiments, HspB1 variants were expressed in M9 minimal medium containing ^13^C-labeled d-glucose as the only carbon source and ^15^N-labeled ammonium chloride as the only nitrogen source and purified as described, with a final size exclusion chromatography (SEC) (Superdex 75, GE Healthcare) step for exchange into the NMR buffer. Concentrations were determined by using a bicinchoninic acid assay (Thermo Pierce).

Full-length human HspB1 (WT or 3P, containing mutations S15D, S78D, and S82D) encoded in the pETHSUL vector with an N-terminal SUMO and 6× His tag was transformed into *E. coli* BL21(DE3) cells. Cells were used to inoculate a 2-ml LB culture and grown for 7 hours at 37°C. Next 500 μl of this culture was transferred to 1 liter of ZYP-5052 autoinduction medium. Cells were left to grow overnight at 24°C and 180 rpm until reaching an optical density (OD) of 4, and then another 24 hours at 18°C and 180 rpm. Cells were harvested and lysed using a cell press with added protease inhibitor cocktail, spun at 20,000*g* for 20 min, and loaded onto a HisTrap column in 50 mM sodium phosphate, 250 mM NaCl, 12.5 mM imidazole, and 5 mM BME (pH 7.5). Protein was eluted with a gradient to 500 mM imidazole. Eluent was pooled and dialyzed overnight with SUMO hydrolase at 4°C into the loading buffer, and the sample was then passed over a HisTrap column and flow-through was collected. This was concentrated and further purified by SEC using a Superdex 200 16/600 column (GE Healthcare). Purity was assessed by SDS–polyacrylamide gel electrophoresis (PAGE), and protein was concentrated, flash-frozen, and stored in PBS with 1 mM DTT at −80°C. Concentration was determined by absorption at 280 nm.

### Expression and purification of FLNC_d18–21_ constructs

The region of human FLNC comprising domains 18 to 21 (WT, residues 1943 to 2408 from UniProt ID Q14315; ∆I, residues 1943 to 2408 excluding 2163 to 2243) was encoded in a modified pET23a/EEF with C-terminal 6× His and EEF tags. The plasmid was transformed into *E. coli* BL21(DE3) pLysS cells (Agilent). To generate the Mβ construct, mutations E2136R and I2138E were introduced by site-directed mutagenesis. Cells were grown in LB at 37°C until an OD_600_ of 0.6 to 0.8 was reached. Isopropylthiogalactoside was then added to a final concentration of 0.5 mM to induce expression for 16 to 18 hours at 21°C. Cells were resuspended in 300 mM NaCl, 50 mm tris, 20 mM imidazole, and 5 mM BME (pH 7.7) containing protease inhibitor cocktail and lysed using a cell press. Lysate was clarified by centrifugation at 20,000*g* for 20 min, filtered through a 0.22-μm filter, and applied to a 5-ml HisTrap column (GE Healthcare). Protein was eluted with a gradient to 500 mM imidazole, pooled, concentrated using an Amicon centrifugal concentrator with a molecular weight cutoff of 30 kDa, and further purified by SEC using a Superdex 200 (10/300) column (GE Healthcare) at 4°C equilibrated in MS buffer [200 mM ammonium acetate (pH 6.9)]. Purity was assessed by SDS-PAGE, and protein was stored in MS buffer at −80°C and used for experiments within 2 weeks of purification, after noticing in initial preparations some propensity for destabilization and aggregation following long-term storage.

### Expression and purification of FLNC_d20–21_ constructs

Cloning of the FLNC_d20–21_ constructs was performed as described previously (*57*) using the p3NH expression vector carrying an N-terminal 6× His tag. FLNC_d20–21_ encompassed residues 2132 to 2406 and ^∆β^FLNC_d20–21_ encompassed residues 2155 to 2406. For protein expression, 6 × 500 ml autoinduction medium (no trace metal mix added) supplemented with chloramphenicol (34 μg ml^−1^) and kanamycin (50 μg ml^−1^) was inoculated with 20 ml of overnight culture and grown at 37°C to OD_600_ of 0.8 shaking at 150 rpm. Then, the temperature was reduced to 20°C, and after 12 hours, the culture was centrifuged (4°C, 45,000*g*) and the resulting cell pellet was either processed immediately or frozen in liquid nitrogen and stored at −80°C.

Cell pellets were resuspended in 100 ml of lysis buffer [1× PBS, 20 mM imidazole, 2 M urea, (pH 7.4)] supplemented with 50 μl of deoxyribonuclease I (10 mg ml^−1^) and sonicated twice for 3 min (50% amplitude; 1 s pulse on; 1 s pulse off, Branson Sonifier W-450 D). The lysate was clarified by centrifugation, and the supernatant was subsequently loaded onto a 5-ml HisTrap FF crude (GE Healthcare) column pre-equilibrated with lysis buffer. Protein was eluted with a step gradient using elution buffer [1× PBS, 500 mM imidazole, 2 M urea (pH 7.4)]. Individual fractions of purified protein were frozen in liquid nitrogen without concentrating and stored at −80°C until further use. Immediately before experiments, protein was exchanged into MS buffer [200 mM ammonium acetate (pH 6.9)] using a Superdex 200 (10/300) column (GE Healthcare) at 4°C. Concentration was measured by absorbance at 280 nm, and the protein was diluted to a concentration of 5 μM for experiments.

### Peptides

Peptides were purchased from Biomatik at 99+% purity with acetylated N termini when the N-terminal residue was Glu. Lyophilized peptides were resuspended in ddH_2_O to a stock concentration of 15 mM, flash-frozen, and stored at −20°C until use.

### Crystallization and structure determination

Crystals were obtained following initial screens using HspB1_84–170_ (18 mg ml^−1^) with a fourfold molar excess of one of several peptide mimics of the N-terminal region varying in length and degree of phosphorylation. Several combinations yielded reproducible crystals but most exhibited poor diffraction (>7 Å). Following rounds of optimization using hanging-drop plates (VDX; Hampton Research), we collected a dataset from a crystal grown from 5 mM peptide ^P82^HspB1_76–88_ and HspB1_84–170_ (16 mg ml^−1^) that allowed for structure determination (table S1). Drops were set using a protein-peptide mixture in PBS (pH 7.0) and equilibrated against 22% polyethylene glycol (PEG) 3350, 0.02 M sodium potassium diphosphate, and 0.1 M bis-tris propane (pH 7.5). The protein was mixed 1:1 with the crystallization solution (0.9 μl each), and drops were left to equilibrate at room temperature against a 1-ml reservoir. Crystals grew within 2 days and continued growing to maximum size within 6 days. Crystals were cryoprotected by serial transfer from the drop to the crystallization solution containing additional 10% PEG 400 and 5% followed by 10% glycerol, before being flash-frozen in liquid N_2_. Diffraction data were collected under cryogenic conditions on beamline I04-1 at Diamond Light Source (Harwell, UK).

Diffraction data were integrated using iMosflm and scaled and merged using Aimless in the CCP4 suite with truncation at 2.1 Å. Phases were determined by molecular replacement using Phaser with a previous HspB1 x-ray structure input as a search model (PDB 4MJH, modified by removal of flexible loops, bound C-terminal peptide, and β2 strands). The four dimers in the asymmetric unit were completed through cycles of manual building in Coot and refinement in Phenix imposing noncrystallographic symmetry (NCS) restraints. Peptide density was then apparent in the 2*F*_o_-*F*_c_ and *F*_o_-*F*_c_ maps next to one monomer within each of the four dimers. Peptide fragments were placed through cycles of manual building in Coot and refinement in PHENIX, now without NCS restraints and with treatment of TLS (translation-libration-screw) parameters. Most peptide side chains remained poorly resolved throughout cycles of refinement in contrast to the well-resolved core. In addition, the peptide density never extended beyond a length accommodating 3 to 6 of 11 residues.

Thus, we attempted to deduce the orientation and identity of the residues captured in the density. In light of data suggesting an intramolecular interaction, we treated each peptide as though extending toward the β2 strand of the monomer to which it is bound. The question of which residues occupy the binding pocket was thus restricted to those that could reach from the β2 strand if the chain was continuous, eliminating the latter half of the peptide and leaving, roughly, ALSRQL. The lack of density for the remaining half of the peptide is unsurprising, because most of these residues are repeated in the ACD and would compete for contacts with their counterparts that are covalently bound to the core and exist at a much higher local concentration, leaving this portion of the peptide no site to bind and thus too disordered to give rise to observable electron density. In each peptide region, the clearest side-chain electron density pointed into a pocket between Phe^104^ and Gly^161^ that could not accommodate a bulky residue but must be larger than alanine. Backbone density extends two residues N-terminally from this pocket, excluding Leu^77^ and implicating Ser^78^ or Leu^81^. Modeling each of these and refining caused negligible difference in *R*_work_ and *R*_free_; therefore, based on close examination of the resulting *F*_o_-*F*_c_ maps and potential chemical contacts within the pocket, as well as the NMR data supporting dynamic changes in this region of the ACD upon Ser^78^ mutation, we placed Ser^78^ into this location. At each dimer interface, Cys^137^ density evidenced both disulfide-bonded and reduced conformations, and so we modeled each of these with occupancy 0.5 in all cases, consistent with the labile nature of this bond (*29*). The final model had *R*_work_/*R*_free_ of 21%/25%.

### NMR spectroscopy

All NMR spectroscopy experiments were recorded at 298 K on a 14.1-T Varian INOVA spectrometer equipped with a 5-mm *z*-axis gradient triple resonance room temperature probe. Spectra were processed with NMRPipe and analyzed with NMRFAM-Sparky. Two-dimensional sensitivity-enhanced ^1^H-^15^N heteronuclear single-quantum coherence (HSQC) spectra were acquired with ^1^H (^15^N) 512 (64) complex points, spectral widths of 8012 Hz (1800 Hz), maximum acquisition times of 64 ms (35.6 ms), an interscan delay of 1 s, and four scans per free induction decay for a total acquisition time of 19 min. ^13^C,^15^N-^2P^HspB1_77–171_ was prepared at a final concentration of 0.9 mM in 30 mM sodium phosphate, 2 mM EDTA (pH 7.0) for resonance assignments, wherein HNCA, HNCO, and HN(CA)CO spectra were recorded. The assigned ^1^H^N^, ^15^N, ^13^CO, and ^13^Cα chemical shifts were analyzed with TALOS-N and RCI to respectively estimate the secondary structure and N-H order parameters.

Protonated, ^15^N-labeled proteins were prepared at 0.9 mM (^2P^HspB1_77–171_) or 1 mM (HspB1_84–171_) for ^15^N relaxation studies. Standard pulse sequences to measure transverse relaxation times (*T*_2_), ^15^N heteronuclear nuclear Overhauser enhancements (hetNOE), and ^15^N CPMG (Carr-Purcell-Meiboom-Gill sequence) relaxation dispersion were used. The *T*_2_ experiments contained eight delay times up to 154 ms and an interscan delay of 2 s. The intensity changes over time (*I*/*I*_0_) were fit to an exponential decay, and values are reported as rates (i.e., 1/*T*_2_) for convenience. hetNOE experiments contained an interscan delay of 8 s, with values representing the intensity upon amide proton saturation divided by the intensity in its absence. CPMG relaxation dispersion experiments were recorded with variable delays between 180° pulses in the CPMG pulse train (τ_CPMG_ = 4ν_CPMG_^−1^). A constant relaxation delay of 39 ms for the CPMG period and 20 ν_CPMG_ values ranging from 54 to 950 Hz were used. Peak shapes were fit with FuDA to extract peak intensities, which were then converted into *R*_2,eff_ values using the following relation: *R*_2,eff_(ν_CPMG_) = −1*/T*_relax_ ln[*I*(ν_CPMG_)/*I*(0)], where *I*(ν_CPMG_) is the intensity of a peak at ν_CPMG_, *T*_relax_ is the constant relaxation delay of 39 ms that was absent in the reference spectrum, and *I*(0) is the intensity of a peak in the reference spectrum. Two duplicate ν_CPMG_ points were recorded in each dispersion dataset for error analysis, and uncertainties in *R*_2,eff_ were calculated using the SD of peak intensities from such duplicate measurements. From plots of *R*_2,eff_ as a function of ν_CPMG_, *R*_ex_ was determined by taking the difference of *R*_2,eff_ (54 Hz) and *R*_2,eff_ (950 Hz) with error bars representing the propagated errors in *R*_2,eff_.

### Native IM-MS

Because HspB1 can form disulfide-linked dimers, experiments involving the ACD were performed in the presence of 10-fold molar excess DTT. Gold-plated capillaries were prepared in-house. Native MS and IM-MS data were recorded on a Synapt G1 mass spectrometer (Waters) modified for the transmission of intact noncovalent protein complexes. HspB1_77–171_ variants were analyzed at a concentration of 10 μM in 200 mM ammonium acetate (pH 6.9). Spectra of FLNC_d18–21_ and peptide titrations were recorded with the following settings: capillary, 1.50 kV; sample cone, 30 V; extractor cone, 3 V; backing pressure, 3.8 mbar; trap gas (argon), 4 ml min^−1^; trap and transfer cell voltages, 5 V. FLNC concentration was held constant at 5 μM, and peptide concentration (HspB1_80–88_ or ^P^HspB1_80–88_) varied from 5 to 160 μM. Peptides and protein were mixed in 200 mM ammonium acetate (pH 6.9) immediately before data collection. Nanoelectrospray ionization was performed in the presence of acetonitrile vapor to promote charge reduction and maintenance of noncovalent contacts and native structure. A minimum of three technical repeats were collected at each ratio. *K*_D_s were obtained by fitting the titration data using a standard binding model of three equivalent binding sites implemented in UniDec software (*58*). Contour plots were generated using a custom script to minimally smooth the data, sort it into 5 *m*/*z* bins, and normalize intensity across the input spectra. To control for nonspecific adduct formation during desolvation, spectra were collected using an excess of HspB1_80–88_ peptide with scrambled sequence. To control for the possibility of promiscuity in recognition of Ig domain folds by HspB1 flexible termini, spectra were collected using an excess of peptide mimicking HspB1 C-terminal residues 177 to 186.

Gas-phase unfolding experiments were performed using the same conditions as described for peptide titrations, with an ion mobility cell gas pressure of 0.53 mbar (N_2_) and a flow rate of 22 ml min^−1^, at a wave velocity of 300 m s^−1^ and a wave height of 7.0 V. Collision voltage into the trap cell varied from 5 to 180 V or until signal was no longer present, in 5-V increments below 100 V, and in 10-V increments above 100 V. All data shown here for complexes were performed with 16-fold molar excess of peptide, excepting controls in fig. S4 as described in the figure legend. Plots were visualized in PULSAR (*59*) and are representative of experiments performed with three different protein batch preparations and three peptide-to-protein ratios. To extract activation thresholds for transitions as indicated in Fig. 4 and figs. S6 and S7, the Unfolding Extraction/Analysis tool in PULSAR was used. To quantify peptide dissociation with activation, the 13+ singly bound peak was selected in the quadrupole with low-mass and high-mass resolutions of 7.2 and 6.9, respectively, and trap voltage ramped as described for the unfolding experiments. Data were smoothed minimally in MassLynx, and peak intensities were extracted, summed as bound or unbound, and converted to percentages. A subtraction was applied to correct for nonspecific adduction at potentials below 30 V based on activation trials with nonspecific controls.

### Calculation of CCSs

CCSs (Ω) of models from the PDB (trimmed in PyMOL as described in the Supplementary Materials) were calculated by the projection approximation method using IMPACT (*60*), accounting for N_2_ as the collision gas. To calculate CCSs of FLNC_d18–21_, native mass spectra of β-lactoglobulin and avidin (Sigma) were collected under identical drift cell conditions (as described for titration experiments, with the mobility wave height lowered to 5.5 V) and used to construct a calibration in PULSAR, which had *R*^2^ > 0.96, using a power law linear fit.

### Visualization of protein structures

All renderings were generated in PyMOL using structures as described in figure legends. To construct the FLNC_d20–21_ model in fig. S6A, FLNA_d20–21_ was isolated from PDB file 2J3S and used as a template for the sculptor tool in PHENIX, with the FLNC sequence threaded onto the FLNA structure.

### SDS-PAGE aggregation assay

HspB1 (WT or 3P) was mixed with FLNC_d18–21_ at a 2:1 ratio (80 and 40 μM, respectively) in 200 mM ammonium acetate (pH 6.9) with 500 μM DTT and incubated at 25°C for 90 min. Aliquots were removed periodically and pelleted at 13,000*g* at 10°C for 10 min. Soluble fractions were diluted threefold with 200 mM ammonium acetate for analysis by SDS-PAGE, and pellets were resuspended in the same volume of 200 mM ammonium acetate and solubilized upon addition of the SDS-PAGE loading buffer. Gel bands were quantified by densitometry, using the raw intensities of FLNC and HspB1 bands to extract relative soluble and insoluble proportions at each time point.

### MD simulations

Two simulations of the HspB1 dimer were prepared using a previous ACD crystal structure (PDB: 4MJH). The system was immersed into a TIP3P water box and neutralized with 0.15 M Na^+^ and Cl^−^ ions. Simulations used the AMBER99SB force field and NAMD2.9 MD engine, with the SHAKE algorithm constraining heavy atoms distances and particle mesh Ewald for treating the electrostatic interactions in periodic boundary conditions. The systems were first minimized with 2000 conjugate gradient steps and subsequently simulated using a 2-fs time step. Systems were equilibrated in the nPT ensemble for 0.5 ns, with a 10 kcal mol^−1^ constrain on protein alpha carbons. Values of 300 K and 1 atm were imposed by Langevin dynamics, using a damping constant of 1 ps^−1^, a piston period of 200 fs, and a piston decay of 50 fs. Constraints were subsequently removed, and systems were simulated in the nVT ensemble at 300 K for 1 ns. Last, production runs were conducted in the nPT ensemble (300 K and 1 atm) for 1 μs. We extracted the coordinates of each individual monomer every 0.1 ns, yielding 1000 frames per monomer. We calculated the order parameter (*S*^2^ of N-H bond vectors) of the datasets independently with a Python code developed in house.

### Statistical analysis

All data are presented as the mean of at least three independent experiments, with error bars as defined in figure legends for Figs. 2 and 4 and fig. S3.

## Supplementary Material

http://advances.sciencemag.org/cgi/content/full/5/5/eaav8421/DC1

Download PDF

## SUPPLEMENTARY MATERIALS

Supplementary material for this article is available at http://advances.sciencemag.org/cgi/content/full/5/5/eaav8421/DC1 Fig. S1. FLNC and HspB1 are up-regulated and partially colocalize in mechanically challenged mouse heart. Fig. S2. Phosphorylation of HspB1 modulates the interaction with FLNC_d18–21_. Fig. S3. Solution NMR of truncated HspB1 variants reveals changes in the dynamics of HspB1 ACD residues upon phosphomimicry. Fig. S4. A crystal structure of the HspB1 ACD in complex with a peptide mimic of N-terminal residues reveals conformational heterogeneity of the PPR and β2 strand. Fig. S5. ^(P)^HspB1_80–88_ binds FLNC_d18–21_ specifically with phosphorylation modifying the extension of the complex. Fig. S6. Coulombically steered unfolding can prompt biologically relevant transitions within FLNC. Fig. S7. Stabilization of an intermediate FLNC unfolding state by HspB1 phosphopeptide is observed across charge states and instrument conditions. Table S1. Data collection and refinement statistics for HspB1_84–170_ in complex with PPR peptide. Table S2. Guide to nomenclature for recombinant proteins and synthetic peptides used in this study.
