# Supplementary material for: HspB1 phosphorylation regulates its intramolecular dynamics and mechanosensitive molecular chaperone interaction with filamin C
Source: Sci Adv. 2019 May 22;5(5):eaav8421. doi: 10.1126/sciadv.aav8421 (PMC6530996; doi:10.1126/sciadv.aav8421)
Supplement: http://advances.sciencemag.org/cgi/content/full/5/5/eaav8421/DC1 [file supp_5_5_eaav8421__index.html]

Science Advances | Science Advances

## Supplementary Materials

**This PDF file includes:**

- Fig. S1. FLNC and HspB1 are up-regulated and partially colocalize in mechanically challenged mouse heart.
- Fig. S2. Phosphorylation of HspB1 modulates the interaction with FLNCd18–21.
- Fig. S3. Solution NMR of truncated HspB1 variants reveals changes in the dynamics of HspB1 ACD residues upon phosphomimicry.
- Fig. S4. A crystal structure of the HspB1 ACD in complex with a peptide mimic of N-terminal residues reveals conformational heterogeneity of the PPR and β2 strand.
- Fig. S5. (P)HspB180–88 binds FLNCd18–21 specifically with phosphorylation modifying the extension of the complex.
- Fig. S6. Coulombically steered unfolding can prompt biologically relevant transitions within FLNC.
- Fig. S7. Stabilization of an intermediate FLNC unfolding state by HspB1 phosphopeptide is observed across charge states and instrument conditions.
- Table S1. Data collection and refinement statistics for HspB184–170 in complex with PPR peptide.
- Table S2. Guide to nomenclature for recombinant proteins and synthetic peptides used in this study.

Download PDF

**Files in this Data Supplement:**

- Adobe PDF - aav8421\_SM.pdf
