# Supplementary material for: HspB1 phosphorylation regulates its intramolecular dynamics and mechanosensitive molecular chaperone interaction with filamin C
Source: Sci Adv. 2019 May 22;5(5):eaav8421. doi: 10.1126/sciadv.aav8421 (PMC6530996; doi:10.1126/sciadv.aav8421)
Supplement: Download PDF [file aav8421_SM.pdf]

Science Advances
AAAS

advances.sciencemag.org/cgi/content/full/5/5/eaav8421/DC1

Supplementary Materials for

HspB1 phosphorylation regulates its intramolecular dynamics and mechanosensitive molecular chaperone interaction with filamin C

Miranda P. Collier, T. Reid Alderson, Carin P. de Villiers, Daisy Nicholls, Heidi Y. Gastall, Timothy M. Allison, Matteo T. Degiacomi, He Jiang, Georg Mlynek, Dieter O. Fürst, Peter F. M. van der Ven, Kristina Djinovic-Carugo, Andrew J. Baldwin, Hugh Watkins, Katja Gehmlich*, Justin L. P. Benesch*

*Corresponding author. Email: justin.benesch@chem.ox.ac.uk (J.L.P.B.); k.gehmlich@bham.ac.uk (K.G.)

Published 22 May 2019, *Sci. Adv.* **5**, eaav8421 (2019)
DOI: 10.1126/sciadv.aav8421

This PDF file includes:

Fig. S1. FLNC and HspB1 are up-regulated and partially colocalize in mechanically challenged mouse heart.
Fig. S2. Phosphorylation of HspB1 modulates the interaction with $FLNC_{d18–21}$.
Fig. S3. Solution NMR of truncated HspB1 variants reveals changes in the dynamics of HspB1 ACD residues upon phosphomimicry.
Fig. S4. A crystal structure of the HspB1 ACD in complex with a peptide mimic of N-terminal residues reveals conformational heterogeneity of the PPR and β2 strand.
Fig. S5. $^{(P)}HspB1_{80–88}$ binds $FLNC_{d18–21}$ specifically with phosphorylation modifying the extension of the complex.
Fig. S6. Coulombically steered unfolding can prompt biologically relevant transitions within FLNC.
Fig. S7. Stabilization of an intermediate FLNC unfolding state by HspB1 phosphopeptide is observed across charge states and instrument conditions.
Table S1. Data collection and refinement statistics for $HspB1_{84–170}$ in complex with PPR peptide.
Table S2. Guide to nomenclature for recombinant proteins and synthetic peptides used in this study.

Supplementary Figures

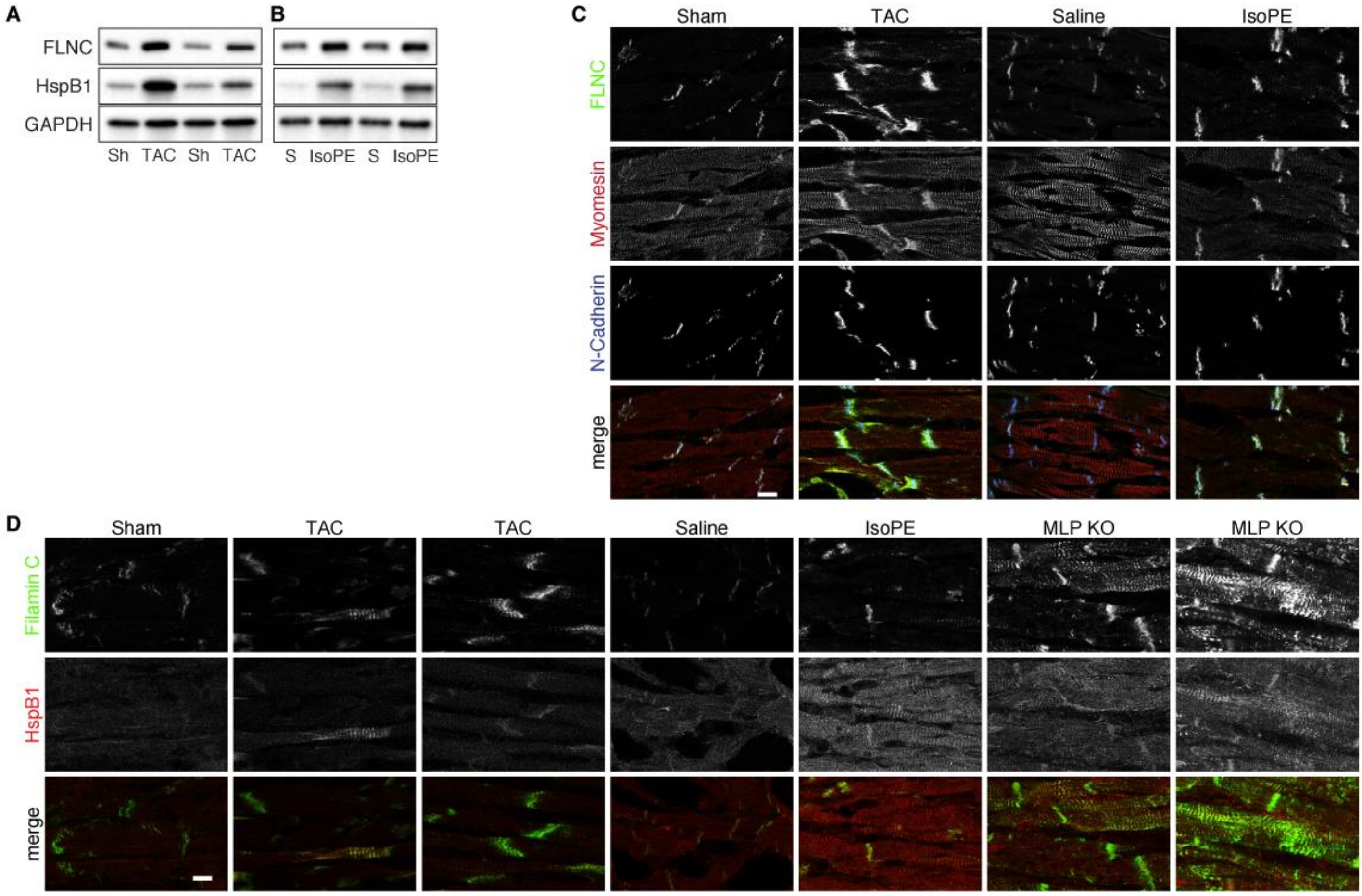

Fig. S1. FLNC and HspB1 are up-regulated and partially colocalize in mechanically challenged mouse heart. Western blots of FLNC and HspB1, and GAPDH as loading control, in **(A)** TAC and **(B)** IsoPE treated WT mouse hearts compared to sham (Sh) and saline (S) controls. **(C)** Frozen sections of WT mouse ventricular tissue stained to visualize FLNC, sarcomeres (myomesin) and intercalated discs (N-cadherin). FLNC is located at intercalated discs and to a lesser extent Z-discs, with greater fluorescence following TAC and IsoPE treatment compared to controls. **(D)** Ventricular tissue from WT TAC and IsoPE groups as well as MLP KO mice stained to visualize FLNC and HspB1. Repeat images are included to show local variation in the disease models. Scale bar = 10 μM.

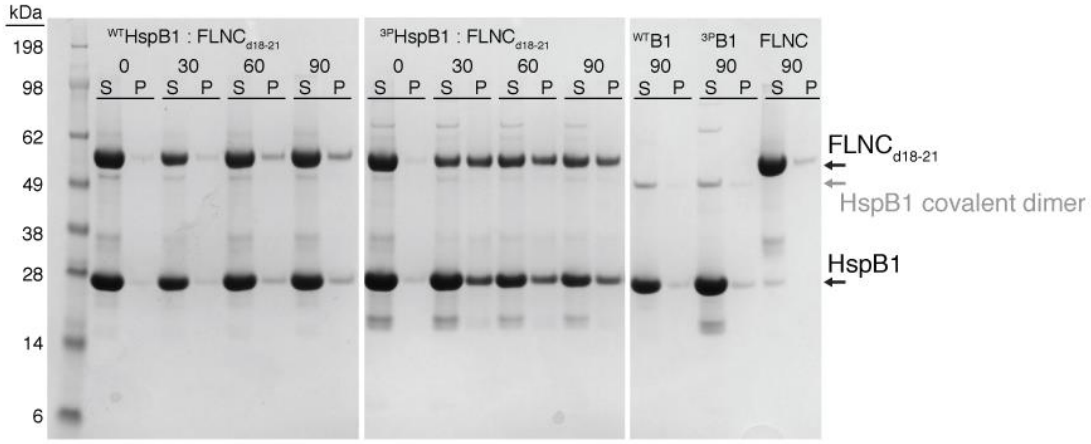

Fig. S2. Phosphorylation of HspB1 modulates the interaction with FLNC$_{d18–21}$. SDS-PAGE aggregation assay. Soluble (S) and pelleted (P) fractions of a 1-to-2 molar ratio mixture of WT (left) or 3P (middle) HspB1 with FLNC$_{d18-21}$ during a 90-minute incubation at 25°C. The gel on the right shows the behavior of the individual proteins without mixing.

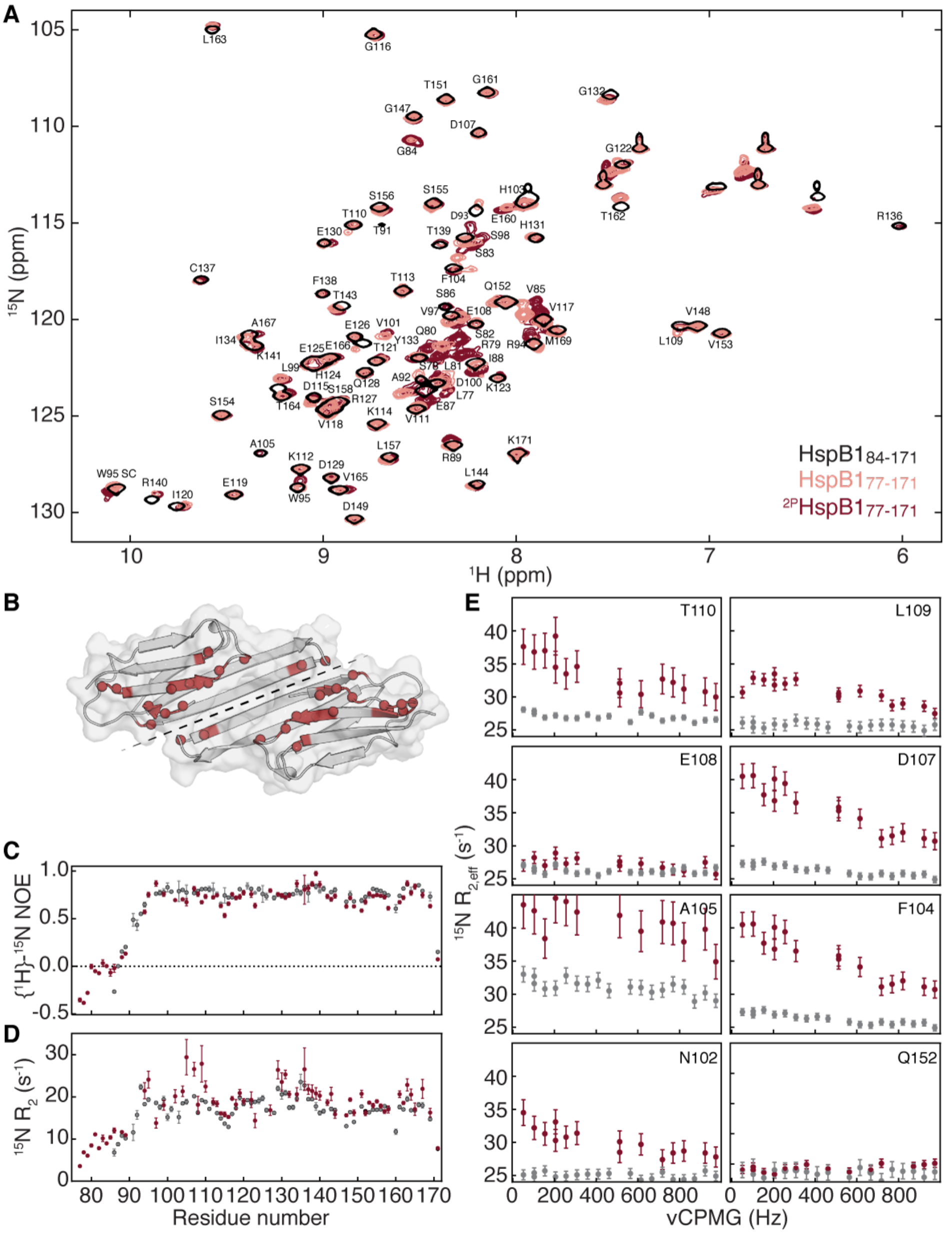

Fig. S3. Solution NMR of truncated HspB1 variants reveals changes in the dynamics of HspB1 ACD residues upon phosphomimicry. (A) ^{1}H-^{15}N HSQC spectra of HspB1$_{84\text{-}171}$, HspB1$_{77\text{-}171}$, and ^{2P}HspB1$_{77\text{-}171}$. Labelled assignments correspond to those from ^{2P}HspB1$_{77\text{-}171}$. Attenuation of signals in HspB1$_{77\text{-}171}$, such as C137, R136, R140, and I120, is likely caused by residual reductant from purification. Intensified signal in the ^{2P}HspB1$_{77\text{-}171}$ sample is likely due in part to locally diminished solvent exchange caused by the Ser to Asp mutations. **(B)** HspB1 ACD (PDB 4MJH) with residues whose chemical shift perturbation (CSP) is greater than 0.1 ppm upon addition of the PPR (with Ser78 and Ser82 mutations) colored. Spheres are backbone nitrogens of affected residues, most of which localize to the β2, β3, or β4 strands. The maximum CSP observed was 0.34 ppm. Dotted line indicates the antiparallel inter-dimer interface. **(C)** ^{1}H-^{15}N heteronuclear NOEs for HspB1$_{84\text{-}171}$ and ^{2P}HspB1$_{77\text{-}171}$ indicate that rapid motions on the ps-ns timescale are similar. HspB1$_{84\text{-}171}$ is shown in gray and ^{2P}HspB1$_{77\text{-}171}$ in maroon. **(D)** ^{15}N transverse relaxation rates (R_2) for HspB1$_{84\text{-}171}$ and ^{2P}HspB1$_{77\text{-}171}$. Elevated ^{15}N R_2 values for ^{2P}HspB1$_{77\text{-}171}$ in residues N102-T110 reflect the presence of conformational exchange on the μs-ms timescale. **(E)** ^{15}N CPMG relaxation dispersion data for individual residues, from which the R_{ex} values in Fig. 2B-C were determined. R_{ex} is defined as the difference in $R_{2,eff}$ values between the lowest and highest ν_{CPMG} effective field strength. In the lower right, Q152 demonstrates the similarity between the two constructs elsewhere in the protein. The colors match those in panels C and D. Data are shown as means ± SD.

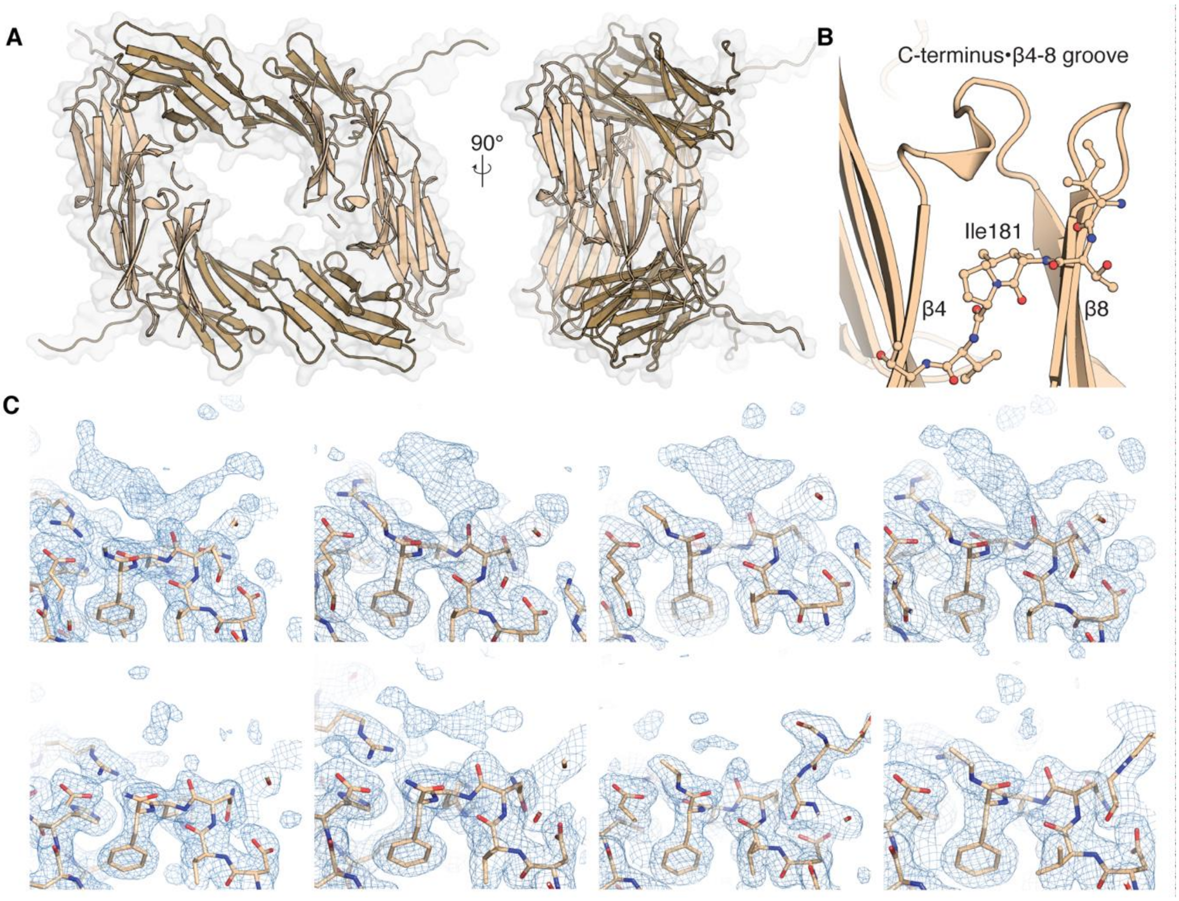

Fig. S4. A crystal structure of the HspB1 ACD in complex with a peptide mimic of N-terminal residues reveals conformational heterogeneity of the PPR and β2 strand. (A) The structure contains four dimers in the asymmetric unit. **(B)** A previous structure (PDB 4MJH), in which a peptide mimic of the - HspB1 C-terminus docks into the groove between strands β4 and β8 via an IPV motif. Ile181 occupies the same binding pocket as Val85 in the new structure (Fig. 3B). **(C)** 2Fo-Fc electron density map contoured at 0.9σ following building and refinement of the ACD prior to placement of any peptides. Top row: N-terminal peptide binding sites for each of the four monomers in the ASU with intramolecularly bound β2 strands; bottom row: equivalent site on each monomer in the same dimer as the image above with extended β2 strands.

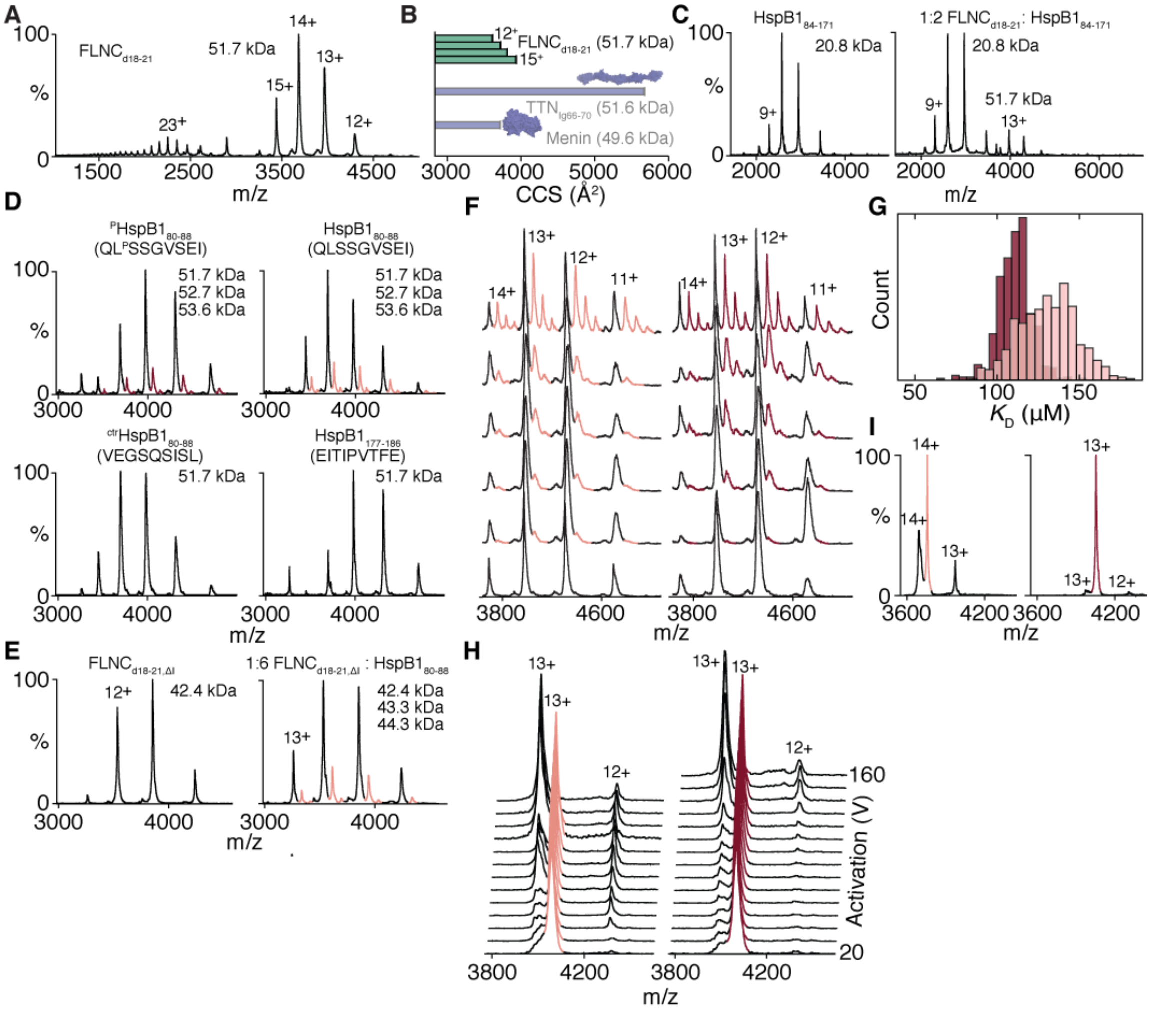

Fig. S5. $^{(P)}$HspB1$_{80–88}$ binds FLNC$_{d18–21}$ specifically with phosphorylation modifying the extension of the complex. (A) Native mass spectrum of FLNC$_{d18-21}$. The high FLNC charge series represents a minor conformation with greater solvent-accessible surface area. **(B)** Green, experimental collision cross section (CCS) measurements of FLNC$_{d18-21}$ charge states 12+ through 15+; blue, theoretical CCS of models derived from the structures of a 50 kDa linear array of Ig domains without inter-domain interactions (titin Ig66-70, trimmed from PDB 3B43) and a 50 kDa globular protein (human menin, PDB 4GQ6) for comparison. FLNC has a substantially lower CCS relative to the prediction for the elongated Ig domains, aligning much more closely to that of the globular protein, evidencing a predominantly highly compact architecture. **(C)** Native mass spectra of HspB1$_{84-171}$ alone and mixed with FLNC$_{d18-21}$. No complex is observed. The most intense charge state in each series is labelled, corresponding to FLNC$_{d18-21}$ and the HspB1$_{84-171}$ dimer. **(D)** Native mass spectra of 5 µM FLNC$_{d18-21}$ with 30 µM peptides: ^{P}HspB1$_{80-88}$; HspB1$_{80-88}$; HspB1$_{80-88}$ with scrambled sequence; HspB1 C-terminal residues 177-186. No appreciable complex is detected with the C-terminal or scrambled peptides, supporting the specificity of N-terminal binding. Bound peaks are shown in color throughout the figure. **(E)** Native mass spectra of FLNC$_{d18-21}$ lacking the d20 insertion (ΔI) individually and mixed 1:6 with peptide HspB1$_{80-88}$. Peptide-bound peaks are observed (pink), evidencing binding to Ig domains rather than the putatively disordered insertion. **(F)** Representative native mass spectra from titration series used to extract binding curves in Figure 4A. 5 µM FLNC$_{d18-21}$ with 5, 10, 20, 40, 80, and 160 µM HspB1$_{80-88}$ (left) and ^{P}HspB1$_{80-88}$ (right). **(G)** Histogram of dissociation constants calculated from the full peptide titration datasets following 500 bootstraps and sorted into 20 bins. The K_D values are 130 ± 17 µM and 110 ± 10 µM for the HspB1$_{80-88}$ and ^{P}HspB1$_{80-88}$ peptides, respectively. These should be regarded as apparent K_Ds and considered in relation to each other rather than as absolute affinities. **(H)** Tandem MS data underlying the breakdown curve in Figure 4C: native mass spectra of 5 µM FLNC$_{d18-21}$ mixed with 80 µM HspB1$_{80-88}$ or ^{P}HspB1$_{80-88}$. The single-peptide-bound 13+ peak was isolated in the quadrupole and activated by acceleration into the collision cell until the complex fully dissociated (140V for HspB1$_{80-88}$, *A*; 160V for ^{P}HspB1$_{80-88}$, *B*). Loss of neutral and singly charged peptide gave rise to 13+ and 12+ unbound peaks. **(I)** Relative peptide bound with activation is independent of peptide concentration and

charge state. Top (5 μM $FLNC_{d18-21}$ with 30 μM $HspB1_{80-88}$ at 80V activation) and bottom (5 μM $FLNC_{d18-21}$ with 160 μM $^{P}HspB1_{80-88}$ at 100V activation) spectra each yielded dissociated fractions within 5% of corresponding activations in **G.**

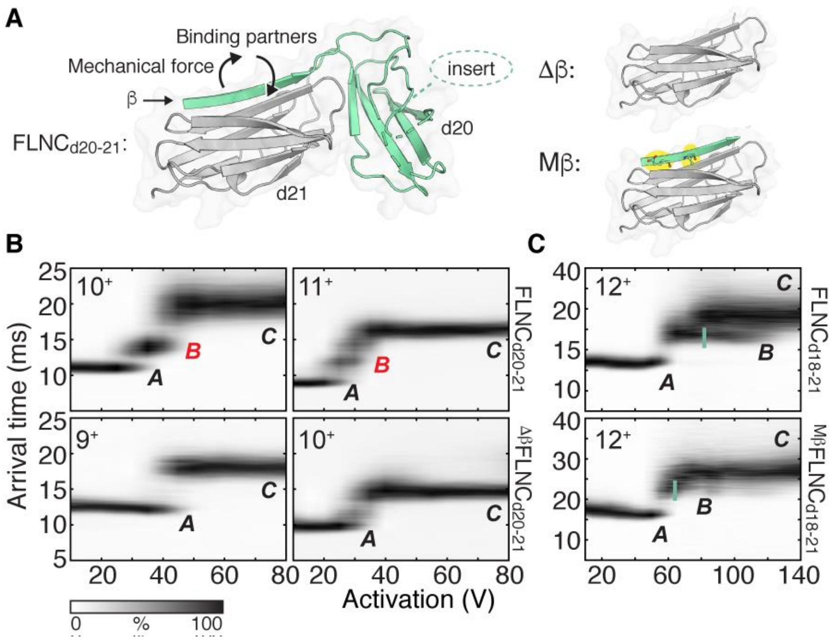

Fig. S6. Coulombically steered unfolding can prompt biologically relevant transitions within FLNC. (A) Partial model of FLNC domains 20-21, which swap a β-strand (β). The 82-residue insertion in FLNC d20 is unstructured, or of unknown structure. At physiological forces, β detaches so that cell adhesion partners can bind d21 (*19*, *33*). We used constructs (right) lacking the strand entirely (Δβ) and containing two residue substitutions to disrupt its binding to d21 (mutated β or Mβ, substitutions highlighted in yellow). **(B)** Coulombic unfolding of $FLNC_{d20\text{-}21}$ and $^{\Delta\beta}FLNC_{d20\text{-}21}$ at two charge states each, selected as most comparable to each other in terms of their charge density. In these terms, $FLNC_{d20\text{-}21}$ 10+ and 11+ (upper panels) are best related to $^{\Delta\beta}FLNC_{d20\text{-}21}$ 9+ and 10+ (lower panels), respectively. Such comparison reveals that removing β abolishes an early unfolding intermediate (red letters). Note that arrival times do not align exactly due to differences in both CCS and charge state of the constructs when containing/lacking the β. **(C)** Coulombic unfolding of mutated β in $FLNC_{d18\text{-}21}$ background, compared to WT. In the larger construct intermediates are less well defined, but effects are still apparent, as the intermediate state *B* is heavily destabilized (lines designate activation required to transition half of *B* to *C*).

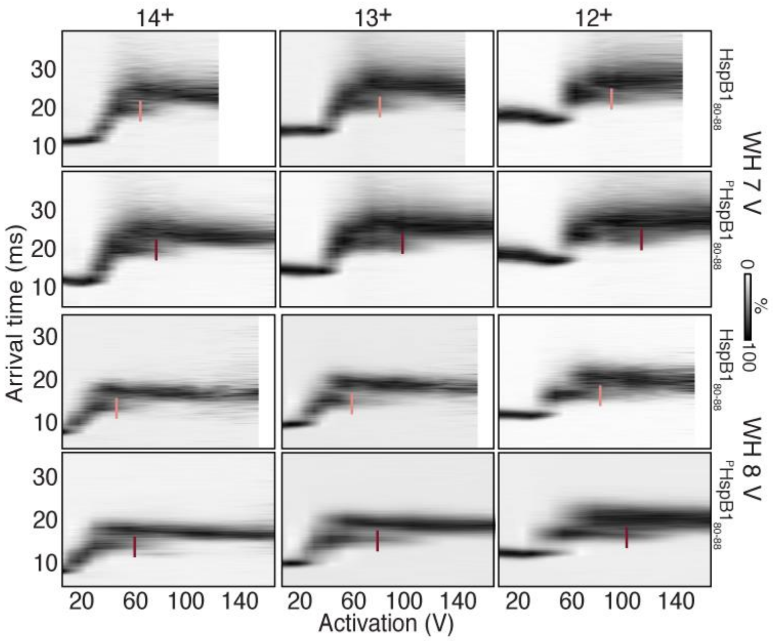

Fig. S7. Stabilization of an intermediate FLNC unfolding state by HspB1 phosphopeptide is observed across charge states and instrument conditions. Coulombic unfolding of $FLNC_{d18-21}$ in complex with HspB1-derived peptides across charge states 14+, 13+, and 12+, and at ion mobility drift cell wave heights (WH) 7.0 V (upper) and 8.0 V (lower). Pink and maroon lines denote voltages required to transition half of the intermediate state to the final state.

Table S1. Data collection and refinement statistics for HspB1$_{84–170}$ in complex with PPR peptide.

Crystal parameters	
Space group	P1211
Cell dimensions	
a, b, c (Å)	56.81, 157.67, 57.87
α, β, γ (°)	90.00, 95.45, 90.00
Dimers in A.S.U.*	4
Data collection	
Synchrotron beamline	DLS I02
Wavelength (Å)	0.9282
Resolution (Å)	78.84-2.10 (2.16-2.10)
Reflections observed/unique	165,497 / 57,271
Redundancy	2.9 (2.9)
Completeness	97.3 (95.8)
Rmeas	0.144 (0.990)
I/σI	5.1 (1.4)
Wilson B-value	31.6
CC1/2	0.99 (0.528)
Refinement	
Resolution (Å)	57.63-2.10 (2.14-2.10)
Reflections	58,913
R_{work}	0.2108
†R_{free}	0.2514
No. of non-H atoms	
Protein	5,622
Water	373
Other	14
Mean B-factors	
Protein (α-crystallin domain)	41.68
Protein (peptide fragments)	84.96
Water	38.23
Other	72.12
RMSD from target values	
Bond length (Å)	0.002
Bond angle (°)	0.524
Ramachandran statistics	
In preferred regions (%)	95.34
In allowed regions (%)	4.51
Outliers (%)	0.15

*Asymmetric unit

†Calculated using 5.14% of reflections

Values in parentheses correspond to the highest resolution shell.

Table S2. Guide to nomenclature for recombinant proteins and synthetic peptides used in this study.

Gene	Protein, construct, region, or peptide	Residues or Domains (d)	Description	Figure(s)
HSPB1	$HspB1^{T7}$	Full-length	Full-length WT HspB1 with an N-terminal T7 tag	1E
	HspB1	Full-length	Full-length WT HspB1	1F, S2
	$^{3P}HspB1$	Full-length	Full-length human HspB1 with Ser to Asp phosphomimic substitutions at residues 15, 78 and 82	1F, S2
	$HspB1_{84\text{-}171}$	84-171	Human HspB1 α-crystallin domain (ACD)	2A, 2B, 2C, S3A-E, S5C
	$HspB1_{84\text{-}170}$	84-170	Human HspB1 ACD excluding residue K171, used for crystallization	3A-C, S4A, S4C
	$HspB1_{77\text{-}171}$ (0P)	77-171	Human HspB1 ACD extended at the N-terminus to encompass the principle phosphorylation region (PPR)	2A, 2D, 2E, S2A
	$^{P}HspB1_{77\text{-}171}$ (1P)	77-171	Human HspB1 ACD and PPR with phosphomimic substitution S82D	2D, 2E
	$^{2P}HspB1_{77\text{-}171}$ (2P)	77-171	Human HspB1 ACD and PPR with phosphomimic substitutions S78E and S82D	2A-E, S2A-E
	$^{P}HspB1_{76\text{-}88}$	76-88	Peptide from human HspB1 phosphorylated at S82, captured in part in crystal structure	3A, 3C, S3A, S3C
	$HspB1_{80\text{-}88}$	80-88	Peptide from human HspB1	4B-D, S5D-I, S7
	$^{P}HspB1_{80\text{-}88}$	80-88	Peptide from human HspB1 phosphorylated at S82	4B-D, S5D, S5F-I, S7
	$^{ctr}HspB1_{80\text{-}88}$	80-88	Peptide containing human HspB1 residues 80-88 in scrambled sequence	S5D
	$HspB1_{177\text{-}186}$	177-186	Peptide from human HspB1	S5D
FLNC	$FLNC_{d18\text{-}21}$	d18-d21	Human FLNC domains 18-21	1E-F, 4B-D, S2, S5A-D, S5F-I, S7
	$FLNC_{d18\text{-}21,\ \Delta I}$	d18-d21	Human FLNC domains 18-21, with 82-residue insertion (I) removed from domain 20	S5E
	$FLNC_{d20\text{-}21}$	d20-21	Human FLNC domains 20-21	S6B
	$^{\Delta\beta}FLNC_{d20\text{-}21}$	d20-21	Human FLNC domains 20-21, with the first β-strand of domain 20 removed	S6B
	$^{M\beta}FLNC_{d18\text{-}21}$	d18-21	Human FLNC domains 18-21, with two mutations (E2136R, I2138E) within the first β-strand of domain 20	S6C
